# Reference ranges for three-dimensional feature tracking cardiac magnetic resonance: comparison with two-dimensional methodology and relevance of age and gender

**DOI:** 10.1007/s10554-017-1277-x

**Published:** 2017-11-27

**Authors:** Boyang Liu, Ahmed M. Dardeer, William E. Moody, Manvir K. Hayer, Shanat Baig, Anna M. Price, Francisco Leyva, Nicola C. Edwards, Richard P. Steeds

**Affiliations:** 1 University Hospital Birmingham NHS Foundation Trust, Birmingham, UK; 2 Minia University, Minia, Egypt; 3 Institute of Cardiovascular Sciences, University of Birmingham, Birmingham, UK

**Keywords:** Three-dimensional feature tracking, Cardiac magnetic resonance, Strain imaging

## Abstract

Myocardial deformation is a sensitive marker of sub-clinical myocardial dysfunction that carries independent prognostic significance across a broad range of cardiovascular diseases. It is now possible to perform 3D feature tracking of SSFP cines on cardiac magnetic resonance imaging (FT-CMR). This study provides reference ranges for 3D FT-CMR and assesses its reproducibility compared to 2D FT-CMR. One hundred healthy individuals with 10 men and women in each of 5 age deciles from 20 to 70 years, underwent 2D and 3D FT-CMR of left ventricular myocardial strain and strain rate using SSFP cines. Good health was defined by the absence of hypertension, diabetes, obesity, dyslipidaemia, or any cardiovascular, renal, hepatic, haematological and systemic inflammatory disease. Normal values for myocardial strain assessed by 3D FT-CMR were consistently lower compared with 2D FT-CMR measures [global circumferential strain (GCS) 3D − 17.6 ± 2.6% vs. 2D − 20.9 ± 3.7%, P < 0.005]. Validity of 3D FT-CMR was confirmed against other markers of systolic function. The 3D algorithm improved reproducibility compared to 2D, with GCS having the best inter-observer agreement [intra-class correlation (ICC) 0.88], followed by global radial strain (GRS; ICC 0.79) and global longitudinal strain (GLS, ICC 0.74). On linear regression analyses, increasing age was weakly associated with increased GCS (R^2^ = 0.15, R = 0.38), peak systolic strain rate, peak late diastolic strain rate, and lower peak early systolic strain rate. 3D FT-CMR offers superior reproducibility compared to 2D FT-CMR, with circumferential strain and strain rates offering excellent intra- and inter-observer variability. Normal range values for myocardial strain measurements using 3D FT-CMR are provided.

**Electronic supplementary material:**

The online version of this article (10.1007/s10554-017-1277-x) contains supplementary material, which is available to authorized users.

## Introduction

Myocardial deformation, as measured through strain and strain rate analysis, is a sensitive marker of sub-clinical myocardial dysfunction that changes before other measures of ventricular performance such as ejection fraction. Global longitudinal strain is an independent predictor of outcome across a broad spectrum of valvular [1] and myocardial [2] diseases, while global circumferential strain provides incremental prognostic value in congenital heart disease [3], as well as predicting ventricular recovery following acute ST-elevation myocardial infarction [4]. Myocardial strain analysis on cardiac magnetic resonance imaging (CMR) has traditionally been performed on 2-dimensional (2D) CMR images, using one of many dedicated deformation sequences such as spatial modulation of magnetization (SPAMM), harmonic phase (HARP), displacement encoding (DENSE) and strain encoding (SENC); or it can be derived from feature tracking of steady-state free precession (SSFP) cine images. This latter technique has been labelled as a “double feature” as it negates the requirement to acquire additional sequences, offers rapid post-processing, while also delivering standard volumetric assessments of the LV [5, 6]. A recent meta-analysis generated normal ranges for feature tracking on CMR (FT-CMR) using the combined data of 659 participants pooled from 18 studies [7]. However, this study also highlighted that previous attempts to define normal range values have employed 2D based techniques which either takes the average of 3 long- or short-axis readings, or more frequently, records a single global longitudinal strain (GLS) value from the 4-chamber view, or a single global circumferential (GCS) and radial (GRS) strain value from the mid LV level. Such 2D based techniques suffer from through-plane loss of features in the third dimension and can be adversely affected by poor tracking within the selected slice which may reduce reproducibility. Furthermore, the assessment of strain from either one or three short-axis (SAX; for circumferential and radial strain) and long-axis (LAX; for longitudinal strain) slices may not be truly representative of global myocardial function. Recently, algorithms have been developed that permit 3D feature tracking of SSFP cine images but there are no data comparing this technique with 2D analysis. The aim of this current study is to determine whether 3D feature-tracking offers superior reproducibility compared to 2D methods and to define the reference ranges for 3D FT-CMR.

## Methods

### Study population

Healthy subjects were originally identified from a prospective, controlled, observational CMR study examining the effects of living kidney donation on cardiovascular structure and function (NCT01028703) [8]. For the purpose of the current study, baseline CMR examinations were included as previously described [5, 9], with the additional recruitment of 15 patients for construction of a cohort of 100 normal healthy subjects in a pre-determined, stratified fashion, to include 10 men and 10 women in each of 5 age deciles from 20 to 70 years. Only individuals in optimal health were included as defined by the absence of hypertension, diabetes, obesity, dyslipidaemia, or any cardiovascular, renal, hepatic, haematological and systemic inflammatory disease. Exclusion criteria included the presence of an abnormal full blood count, serum electrolytes, or resting 12-lead ECG. Demographic data were collected, including height, weight, body surface area, heart rate and office blood pressure (normal < 140/90 mmHg). The study protocol conformed to the ethical guidelines of the 1975 Declaration of Helsinki and written informed consent was obtained from each subject.

### CMR acquisition

CMR studies were conducted using a 1.5-T scanner (Magnetom Avanto, Siemens, Germany). Vertical long axis (VLA) and horizontal long axis (HLA) SSFP cine imaging (retrospective electrocardiographic gating, SSFP) of the left and right ventricles was performed. These images were then used to pilot the LV short axis stack acquired using serial contiguous short axis cines (typical parameters were: resolution 40–50 ms, repetition time 3.2 ms, echo time 1.7 ms, flip angle 60, field of view 300 mm, in-plane resolution 1.5 × 1.5 mm^2^, slice thickness 7 mm with 3 mm gap, minimum 25 phases per cardiac cycle) in accordance with previously validated methodology [10].

### CMR analysis

Analysis of LV function, volume and mass was performed by an experienced operator (BL) with delineation of papillary muscles and trabeculations using thresholding (cvi42^®^ version 5.3.4, Circle Cardiovascular Imaging, Canada). Measurements were made off-line using the contiguous short axis multi-slice acquisition with delineation of atria/ventricles confirmed in matched long axis planes [10]. For ventricular volume analysis, the endocardial border was detected and the largest and smallest cavity volumes were defined as end-diastole and end-systole respectively. The endocardial border was defined as the boundary between blood pool and myocardium, with papillary muscles excluded from volumes. Segmental function was analysed according to a modified version of the American Heart Association 17-segment model [11], with omission of the apical cap.

### Feature tracking CMR

**Fig. 1:**
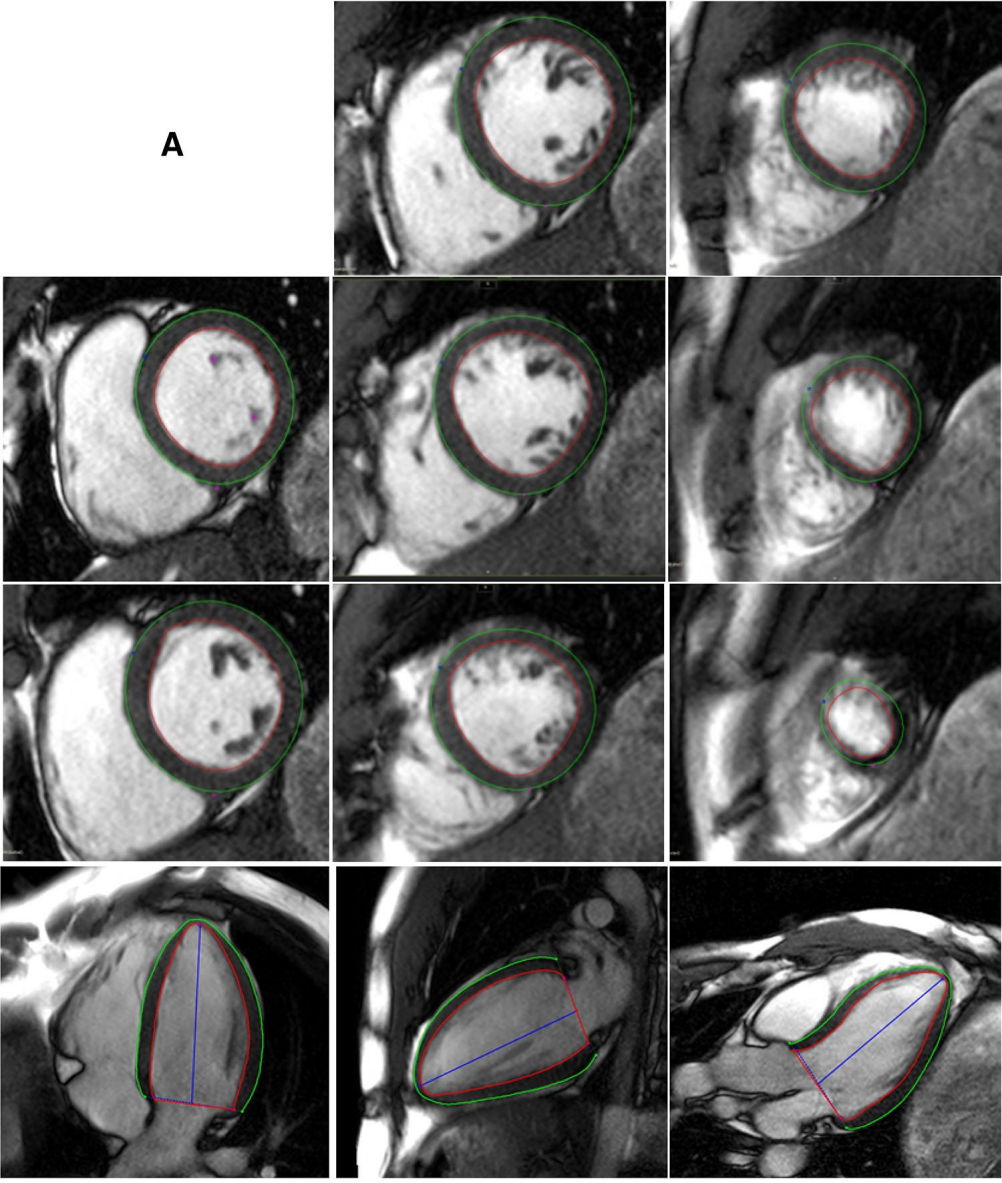

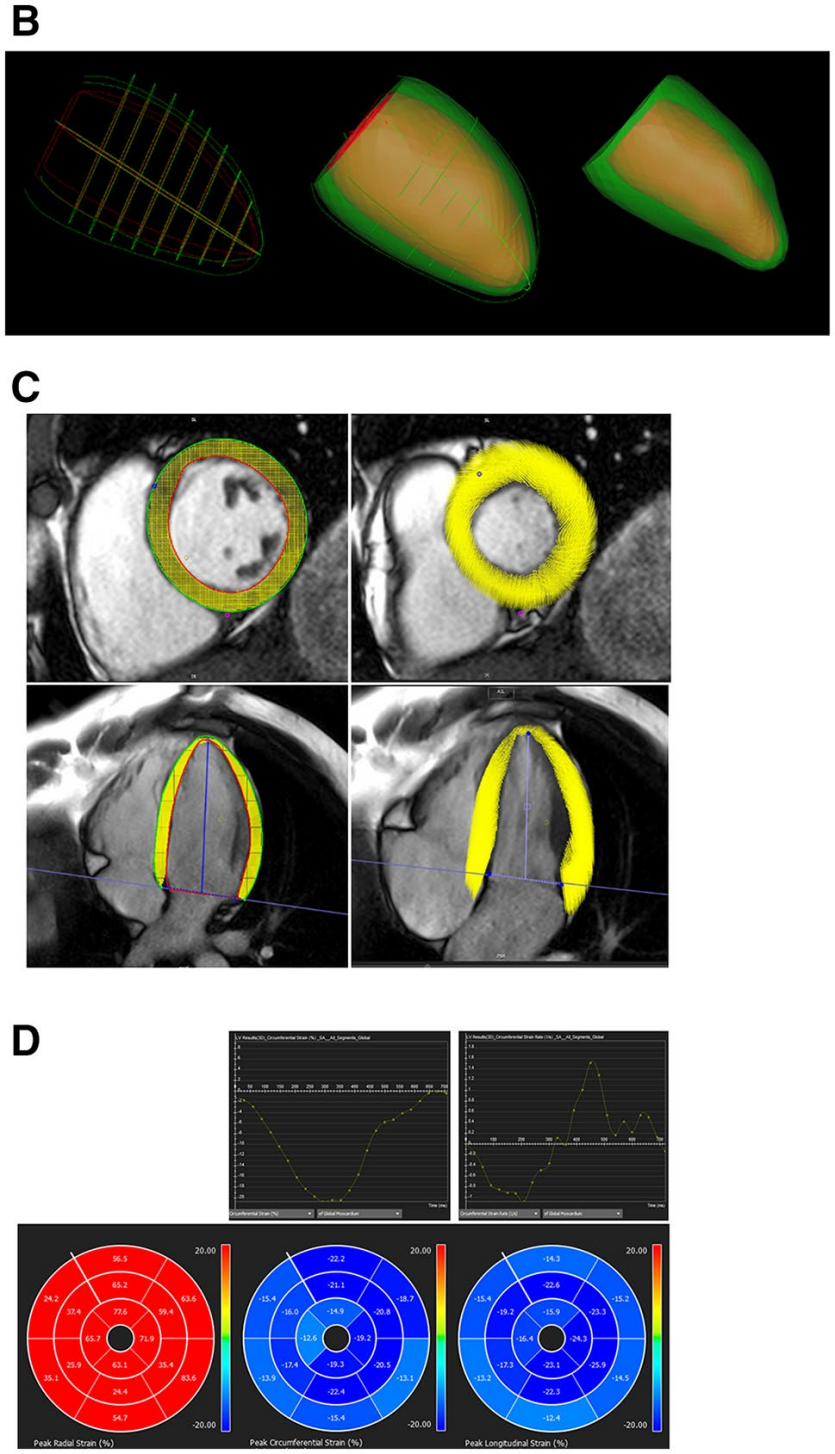
Steps taken for 3D FT-CMR. **a** Define endocardial and epicardial borders. **b** 3D construct of endocardial and epicardial borders are used to generate a 3D model of the myocardium in diastole which is tracked through to systole. **c** Ensure good quality tracking. **d** Results for global and/or segmental strain and strain rates

2D and 3D GCS, GRS, and GLS strain as well as strain rates (S’—peak systolic strain rate; E’—peak early diastolic strain rate; A’—peak late diastolic strain rate) were obtained using cvi42 (version 5.3.4). Smoothed endocardial and epicardial borders were drawn in the end-diastolic frame. For 2D strain analysis, circumferential (Ecc) and radial (Err) strain and strain rates were obtained at the mid LV in the short axis view, 2D longitudinal strain (Ell) and strain rates were obtained from the HLA image [5]. The level of the mid LV was determined and recorded by observer 1 (BL) in order for observer 2 (AD) to replicate analyses at the same level. 3D feature tracking was performed by defining contours in the end-diastolic frame of all short and long axis slices before defining the superior RV insertion points within the LV (Fig. 1).

Feature tracking on cvi42 derives myocardial strains by fitting a nearly incompressible deformable 2D model to individual 2D cine slices over the cardiac cycle. The degree of deformation is determined by a set of imaginary nodes placed on the mid-curve between the endo- and epicardial boundaries and these boundaries are tracked by a pre-defined algorithm (Appendix 1) through the cardiac cycle. Similarly, in 3D feature tracking, a 3D deformable model of the myocardium is generated (Appendix 2) in the end-diastolic phase by interpolating the endo- and epicardial boundaries tracked by the 2D algorithm. The basis of these algorithms has been previously described and their validity demonstrated [12, 13]. The accuracy of feature tracking was manually checked following automated strain analysis on the 2D and 3D CMR models by assessing the tracking of the endocardial and epicardial borders; however, to minimize variability, a maximum of two user adjustments were allowed in the event of significant mis-tracking.

Image quality for each study was rated by observer 1 (BL) assigning a score from 1 to 3 [(1) suboptimal image quality—containing breathing or gating artefacts; (2) average image quality with mild blurring affecting up to 3 cine slices; (3) good image quality with clear endo- and epicardial border delineations throughout the cardiac cycle].

### Reproducibility studies

All CMR studies were anonymised prior to strain analysis. For intra-observer variability, observer 1 (BL) performed feature tracking analyses for all 100 subjects, with a second complete analysis repeated after a 1-month interval in every subject. For inter-observer variability, observer 2 (AD) independently feature tracked a randomly generated set of 45 scans.

### Comparison of strain with other measures of systolic function

To compare the strain results derived from 3D FT-CMR on cvi42, we correlated 3D strain parameters with LVEF and 2D endocardial strains derived from the mid SAX (for Ecc and Err) and HLA (for Ell) cine slices using Diogenes software (TomTec Imaging Systems, Munich, Germany), which offers good agreement with SPAMM myocardial tagging [5].

### Statistical analyses

Data are presented as mean ± standard deviation, median (interquartile range), or frequency (percentage). Data distribution for continuous variables was assessed using normality plots and the Shapiro–Wilk test. Paired t-tests were used to compare the size of biases between 2D and 3D derived strain. The independent samples t-test was used to explore gender differences amongst strain and strain rates. Correlations were assessed with Pearson’s correlation coefficient. One-way ANOVA were used to assess the relationship between image quality and reproducibility bias. Age was treated as a continuous variable within statistical analyses. Linear regression analysis was used to explore the relationship between strain and baseline variables. Variables reaching a P-value of < 0.10 were included in stepwise backward multivariable regression models. Intra- and inter-observer agreement was tested by calculating mean bias and 95% limits of agreement (confidence intervals) from Bland–Altman analyses, and intra-class correlation coefficient (ICC) for absolute agreement. Mean segmental GCS was compared across the 16 segments using a repeated measures ANOVA with Huynh–Feldt adjustment. A P-value of < 0.05 was considered statistically significant. Statistical analysis was performed using SPSS v23.0. (SPSS, Inc., Chicago, IL, USA).

## Results

### Demographics, ventricular volumes and ejection fraction

Baseline demographics are illustrated in Table 1. 85 subjects had a 10-year QRISK-2 score of < 10% and all subjects had a 10-year QRISK-2 score of < 20% [14]. Cardiac volumes, mass and function according to each age decile are listed in Table 2; values were within normal limits for all participants [10]. On linear regression analyses, there were no significant correlations between age and the parameters of height, weight, BSA, or eGFR. Meanwhile, increasing age correlated with increasing LVEF (r = 0.4, P < 0.001), RVEF (r = 0.2, P = 0.03), and decreasing indexed biventricular volumes (LVEDVi r = − 0.4, P < 0.001; LVESVi r = − 0.45, P = < 0.001; RVEDVi r = − 0.3, P = 0.001; RVESVi r = − 0.3, P = 0.001). There was no association between age and indexed LV mass. There were no significant differences between men and women for indexed biventricular volumes or function but men had higher indexed LV mass compared to women (Table 1).

**Table 1:** Baseline demographics of 100 health subjects

	Female (n = 50)	Male (n = 50)	Overall (n = 100)	P
Age (years)	44.8 ± 14.3	44.7 ± 14.3	44.8 ± 14.3	0.98
Height (cm)	163.8 ± 5.6	178.2 ± 8.6	171.2 ± 10.2	< 0.001
Weight (kg)	69.9 ± 11.7	80.9 ± 12.8	75.5 ± 13.4	< 0.001
BSA (m^2^)	1.8 ± 0.2	2.0 ± 0.2	1.9 ± 0.2	< 0.001
LVEF (%)	70.5 ± 6.7	70.8 ± 6.7	70.7 ± 6.7	0.81
LVEDVi (ml/m^2^)	64.1 ± 13.1	65.5 ± 11.6	64.8 ± 12.3	0.57
LVESVi (ml/m^2^)	19.4 ± 7.5	19.6 ± 7.0	19.5 ± 7.2	0.88
LVMi (kg/m^2^)	52.1 ± 9.9	62.9 ± 12.1	57.4 ± 12.2	< 0.001
RVEF (%)	67.5 ± 8.4	66.3 ± 7.1	66.9 ± 7.8	0.46
RVEDVi (ml/m^2^)	63.4 ± 13.2	68.4 ± 14.2	65.8 ± 13.9	0.07
RVESVi (ml/m^2^)	21.0 ± 8.0	23.7 ± 9.5	22.3 ± 8.8	0.14
Haemoglobin (g/l)	13.1 ± 0.8	14.5 ± 1.0	13.8 ± 1.2	< 0.001
eGFR (ml/min)	85.1 ± 13.5	88.8 ± 12.7	86.8 ± 13.2	0.18

*LV* left ventricular, *RV* right ventricular, *EF* ejection fraction, *EDVi* indexed end diastolic volume, *ESVi* indexed end systolic volume, *LVMi* indexed left ventricular mass

**Table 2:** Ventricular volumes and function according to age decile

	Age				
	20–29 years	30–39 years	40–49 years	50–59 years	60–69 years
LVEF (%)	**68** ± **6**	**68** ± **5**	**70** ± **6**	**73** ± **6**	**75** ± **8**
LVEDVi (ml/m^2^)	**71** ± **15**	**69** ± **12**	**66** ± **9**	**62** ± **13**	**57** ± **7**
LVESVi (ml/m^2^)	**23** ± **7**	**22** ± **7**	**20** ± **6**	**17** ± **8**	**15** ± **6**
LVMi (g/m^2^)	52 ± 14	62 ± 13	62 ± 14	56 ± 9	55 ± 9
RVEF (%)	**65** ± **7**	**66** ± **9**	**66** ± **6**	**69** ± **7**	**69** ± **10**
RVEDVi (ml/m^2^)	**73** ± **17**	**68** ± **15**	**67** ± **11**	**59** ± **12**	**62** ± **10**
RVESVi (ml/m^2^)	**26** ± **9**	**24** ± **9**	**24** ± **9**	**18** ± **6**	**20** ± **9**

Parameters in bold denotes significant correlation with age

### Reference values for global strain and strain rate

Good quality tracking was obtained for all subjects following a maximum of two editions. 3D FT-CMR normal range values for the whole cohort are listed in Table 3 and were defined as the 95% confidence interval of the whole cohort regardless of age. The borderline zones were defined as the upper and lower ranges where measured value lay outside the 95% confidence interval for at least one age group. The abnormal zones were defined by the range where measured values lay outside the 95% confidence interval for any age group.

**Table 3:** Reference values for 3D FT-CMR

Abnormally low and high refer to the lower and upper reference limits are defined as measurements which lie outside the 95% confidence interval at all age groups. Borderline zone values should be looked up in the age-specific tables (Table 4(a), (b)). The borderline zone was defined as the upper and lower ranges where the measured value lay outside the 95% prediction interval for at least one age group

Peak strains obtained via 3D feature tracking were lower than corresponding 2D peak strains for GLS and GCS but not for GRS (Supplementary Table 1). Similarly, peak systolic (S’), early diastolic peak (E’) and late diastolic peak (A’) strain rates obtained from 3D were generally lower than those obtained with 2D feature tracking.

### Effect of gender and age

There was no relationship between gender and strain or strain rate, with the exception that E’ was more negative in females than males (Supplementary Table 2). 3D peak strains increased with age, with a more noticeable change after the age of 50, although the correlation was weak (GLS = 12.73 + 0.04 × age, R^2^ = 0.06, R = 24; GCS = 14.52 + 0.07 × age, R^2^ = 0.15, R = 0.38; GRS = 35.07 + 0.28 × age, R^2^ = 0.10, R = 0.32) (Table 4(a)). Increasing age was related to higher peak systolic and late diastolic strain rates (Table 4(b)). Increasing age was also associated with a reduction in early diastolic strain rate for circumferential and longitudinal, but not radial directions. There were no interaction effects between age and gender for the prediction of strain on multivariable regression analyses.

**Table 4:** (a) 3D peak strain across age deciles. (b) Age adjusted 3D peak strain rates and results of linear regression analyses on the relationship between age and strain rate

(a)					
	Strain by age decile (%)				
	20–29 years	30–39 years	40–49 years	50–59 years	60–69 years
GCS	− 16.9 ± 2.1	− 16.8 ± 2.3	− 16.4 ± 2.4	− 18.2 ± 2.0	− 19.7 ± 2.6
GLS	− 14.8 ± 2.1	− 13.4 ± 2.3	− 14.0 ± 2.8	− 14.9 ± 2.2	− 16.2 ± 2.7
GRS	45.9 ± 12.0	43.9 ± 9.8	42.4 ± 10.8	47.9 ± 9.0	57.5 ± 14.7

(b)									
	Strain rate by age decile (1/s)					Statistical analyses			
	20–29 years	30–39 years	40–49 years	50–59 years	60–69 years	R	R^2^	β	P
GCS S’	− 0.87 ± 0.13	− 0.86 ± 0.19	− 0.90 ± 0.23	− 0.92 ± 0.18	− 1.03 ± 0.18	0.26	0.07	0.003	**0.009**
GCS E’	1.06 ± 0.19	0.98 ± 0.24	0.95 ± 0.22	0.91 ± 0.24	0.88 ± 0.20	− 0.26	0.07	− 0.004	**0.008**
GCS A’	0.36 ± 0.09	0.37 ± 0.11	0.44 ± 0.08	0.55 ± 0.12	0.69 ± 0.14	0.74	0.55	0.008	< **0.001**
GRS S’	2.79 ± 0.84	2.63 ± 0.95	2.61 ± 0.96	2.89 ± 0.72	3.80 ± 1.51	0.29	0.09	0.023	**0.003**
GRS E’	− 3.30 ± 1.18	− 2.85 ± 0.90	− 2.85 ± 1.24	− 3.06 ± 0.88	− 3.16 ± 1.02	− 0.12	0.00	− 0.001	0.906
GRS A’	− 0.50 ± 0.12	− 0.52 ± 0.17	− 0.62 ± 0.16	− 0.79 ± 0.23	− 0.97 ± 0.29	0.66	0.43	0.012	< **0.001**
GLS S’	− 0.74 ± 0.12	− 0.69 ± 0.16	− 0.77 ± 0.20	− 0.76 ± 0.15	− 0.82 ± 0.18	0.26	0.07	0.005	**0.009**
GLS E’	0.98 ± 0.26	0.81 ± 0.24	0.90 ± 0.31	0.73 ± 0.16	0.74 ± 0.17	− 0.30	0.01	− 0.005	**0.002**
GLS A’	0.34 ± 0.09	0.34 ± 0.08	0.40 ± 0.09	0.49 ± 0.10	0.59 ± 0.10	0.69	0.47	0.007	< **0.001**

P values < 0.05 are highlighted in bold

### Reproducibility

**Fig. 2:**
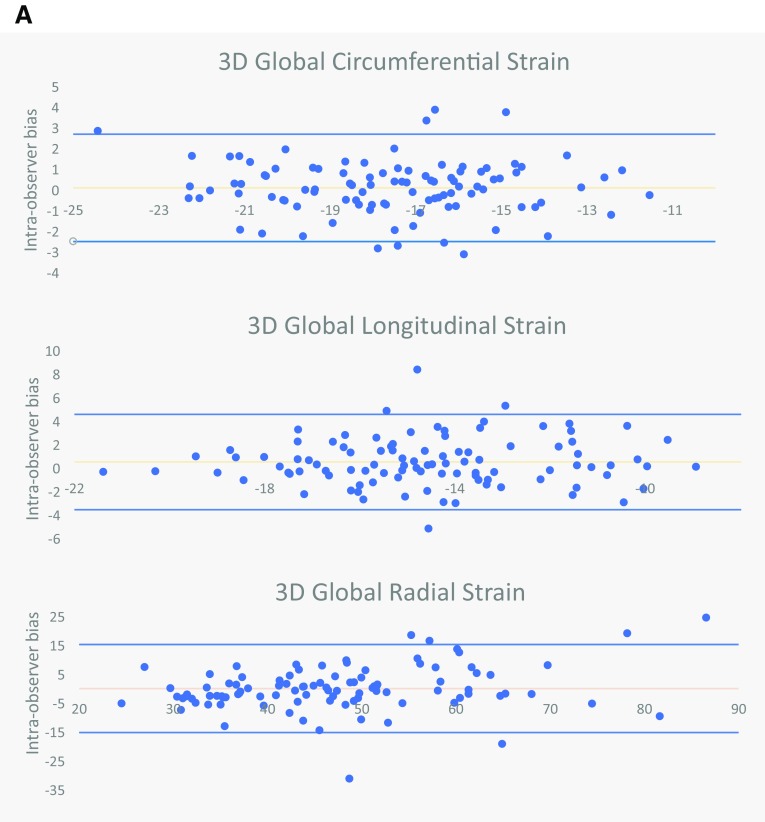

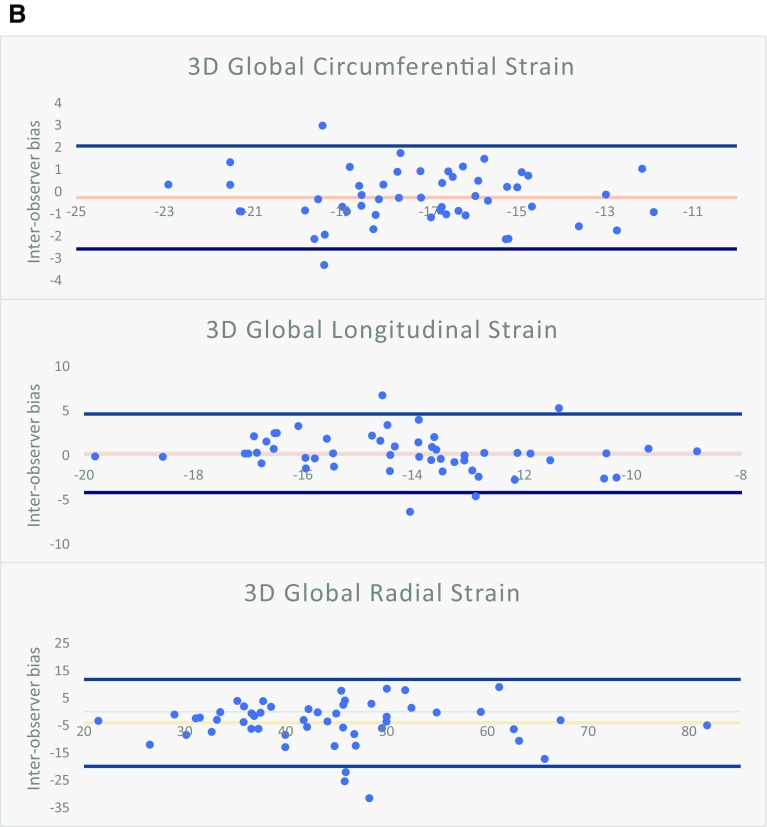
**a** Bland–Altman plots for intra-observer bias for 3D peak GCS, GRS, and GLS. **b** Bland–Altman plots for inter-observer bias for 3D peak GCS, GRS, and GLS

Intra- and inter-observer variability are listed in Table 5; intra- and inter-observer limits of agreement are illustrated in the Bland–Altman analyses of Fig. 2a, b respectively. Reproducibility biases were significantly lower for almost all strain and strain rates when derived from 3D feature tracking models. Similarly, 3D feature tracking had superior ICC compared to 2D models for the majority of peak strain and strain rate parameters. For peak strain, 3D GCS has the highest intra-observer, followed by GRS and GLS. If the mean strain or strain rate of 2 separate analyses were used then significant improvement in ICC could be gained (Supplementary Table 3).

**Table 5:** 2D versus 3D intra- and inter-observer reproducibility for peak strain and strain rates

	Intra-observer reproducibility		Inter-observer reproducibility	
	Mean absolute bias	ICC (95% CI)	Mean absolute bias	ICC (95% CI)
*Circumferential*				
3D GCS	1.04 ± 0.83	0.88 (0.83–0.92)	0.94 ± 0.71	0.88 (0.79–0.93)
2D GCS	1.73 ± 1.52*	0.82 (0.75–0.88)	2.18 ± 1.77*	0.66 (0.45–0.79)
3D GCS S’	0.09 ± 0.09	0.81 (0.73–0.87)	0.09 ± 0.11	0.67 (0.47–0.80)
2D GCS S’	0.26 ± 0.34*	0.44 (0.27–0.59)	0.26 ± 0.27*	0.41 (0.14–0.62)
3D GCS E’	0.16 ± 0.13	0.64 (0.51–0.75)	0.15 ± 0.16	0.72 (0.54–0.84)
2D GCS E’	0.49 ± 0.45*	0.27 (0.08–0.45)	0.52 ± 0.56*	0.00 (− 0.27 to 0.29)
3D GCS A’	0.04 ± 0.05	0.93 (0.90–0.95)	0.04 ± 0.04	0.93 (0.88–0.96)
2D GCS A’	0.13 ± 0.11*	0.74 (0.63–0.82)	0.17 ± 0.13*	0.58 (0.35–0.75)
*Longitudinal*				
3D GLS	1.45 ± 1.21	0.76 (0.66–0.84)	1.29 ± 1.12	0.74 (0.57–0.85)
2D GLS	1.91 ± 1.51Ɨ	0.66 (0.53–0.76)	1.83 ± 1.31^	0.70 (0.52–0.83)
3D GLS S’	0.15 ± 0.16	0.36 (0.18–0.52)	0.11 ± 0.11	0.62 (0.40–0.77)
2D GLS S’	0.20 ± 0.17Ɨ	0.48 (0.31–0.62)	0.18 ± 0.14*	0.62 (0.40–0.77)
3D GLS E’	0.20 ± 0.23	0.50 (0.33–0.63)	0.14 ± 0.15	0.54 (0.30–0.72)
2D GLS E’	0.22 ± 0.19	0.53 (0.37–0.66)	0.23 ± 0.16Ɨ	0.48 (0.22–0.68)
3D GLS A’	0.05 ± 0.06	0.80 (0.71–0.86)	0.05 ± 0.06	0.80 (0.66–0.89)
2D GLS A’	0.17 ± 0.20*	0.66 (0.53–0.76)	0.19 ± 0.18*	0.54 (0.30–0.72)
*Radial*				
3D GRS	5.32 ± 5.55	0.82 (0.75–0.88)	5.18 ± 5.15	0.79 (0.61–0.89)
2D GRS	7.43 ± 8.36*	0.74 (0.63–0.82)	8.38 ± 9.00	0.42 (0.16–0.63)
3D GRS S’	0.56 ± 0.60	0.75 (0.65–0.83)	0.50 ± 0.49	0.73 (0.55–0.85)
2D GRS S’	1.02 ± 1.23*	0.57 (0.24–0.69)	1.31 ± 1.60*	0.14 (− 0.12 to 0.39)
3D GRS E’	0.71 ± 0.63	0.67 (0.54–0.76)	0.65 ± 0.65	0.49 (0.22–0.68)
2D GRS E’	1.29 ± 1.24*	0.42 (0.24–0.57)	1.65 ± 1.31*	0.11 (− 0.10 to 0.35)
3D GRS A’	0.10 ± 0.12	0.85 (0.78–0.90)	0.10 ± 0.09	0.86 (0.76–0.92)
2D GRS A’	0.20 ± 0.22*	0.64 (0.51–0.75)	0.21 ± 0.17*	0.70 (0.51–0.83)

*ICC* intra-class correlation for single measures

Statistical significance: *denotes paired T test P < 0.001, ^denotes P < 0.01, Ɨ denotes P < 0.05 when comparing the size of bias derived from 2D versus 3D feature tracking on paired t-test

### Image quality

The image quality of MRI studies were rated as 1 (suboptimal) in 2 cases, 2 (average) in 11 cases, 3 (good) in 87 cases. There was no relationship between the subjective quality of a CMR study and the size of intra- and inter-observer biases for 2D and 3D data (data not shown).

### Segmental strain

**Fig. 3:**
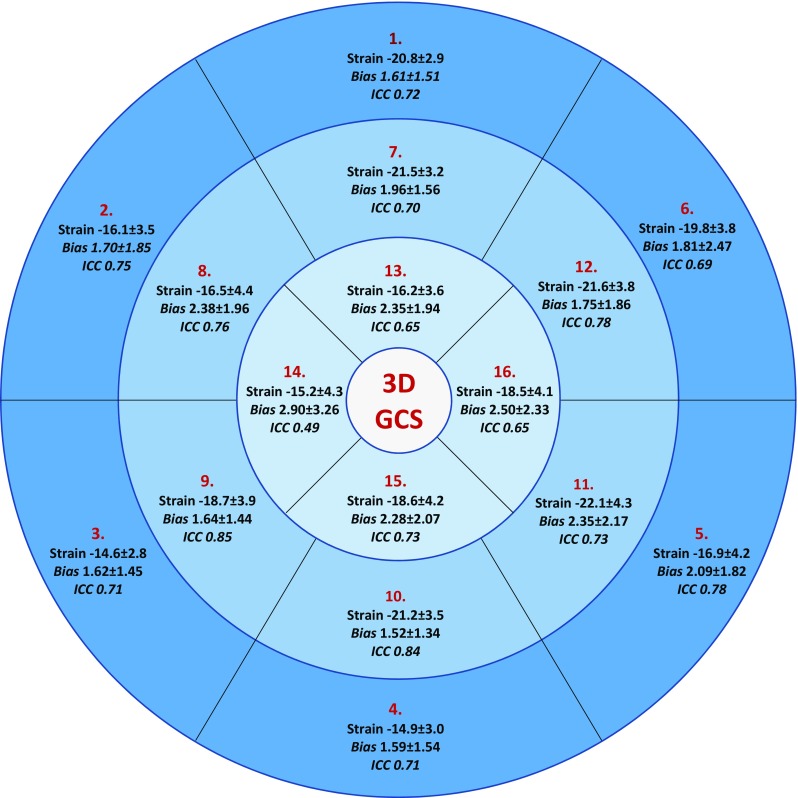
16 segment model illustrating peak GCS ± SD with mean intra-observer absolute bias ± SD and ICC

The normal range values and reproducibility of GCS were calculated when the myocardium was split according to a modified American Heart Association left ventricular model with omission of the apical cap (Fig. 3) [11]. Repeated-measures ANOVA demonstrated significant regional variations in GCS (P < 0.001), with reproducibility being generally good or excellent in the basal and mid segments but lower in the apical segments. Segmental peak strain in the longitudinal and radial direction was poorly reproducible compared to the circumferential direction; these results are illustrated in Supplementary Figs. 1 and 2.

### Comparison of strain with other measures of systolic function

**Fig. 4:**
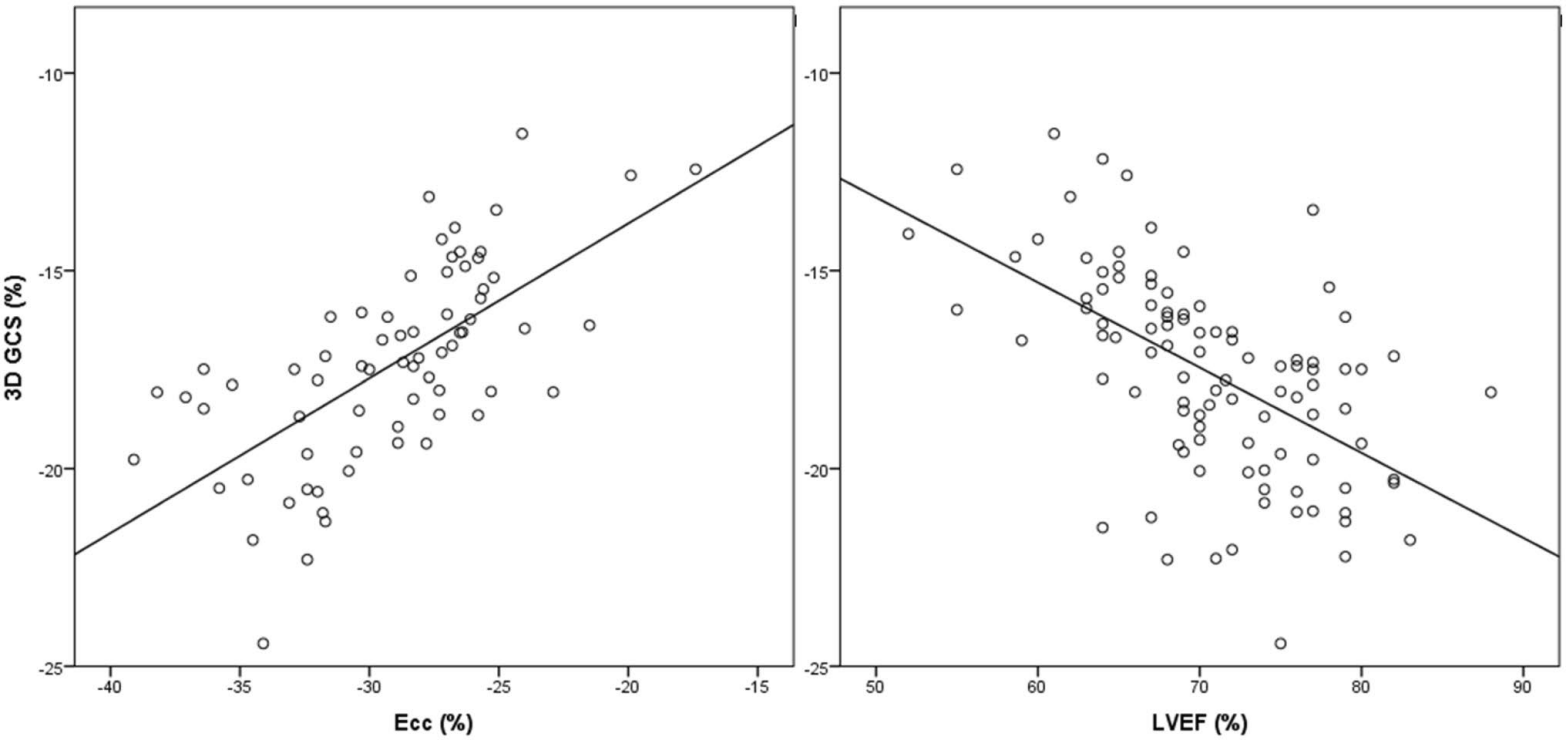
Correlation of 3D GCS against Ecc and LVEF

Cross-platform comparison of peak systolic strain was analysed in a random subset of 70 subjects. Whilst 2D FT-CMR for Ecc (− 29.1 ± 4.2) and Ell (− 24.4 ± 4.8) on Diogenes was significantly higher than 3D strain derived using cvi42 (P < 0.001 for both strain types), there was reasonable correlation between the two algorithms (Fig. 4, circumferential strain r = 0.66 P < 0.001, longitudinal strain r = 0.58 P < 0.001).

Similarly, LVEF was correlated with 3D GCS (Fig. 4, r = 0.56, P < 0.001), GRS (r = 0.60, P < 0.001) and GLS (r = 0.42, P < 0.001). We have not analysed Err on Diogenes due to its lower reproducibility resulting from poor epicardial tracking at the lung and epicardium interface [9].

## Discussion

In this population of age-stratified healthy volunteers, 3D FT-CMR consistently delivered better intra- and inter-observer variability for deformation analysis than the 2D based method. Optimal reproducibility with 3D deformation analysis was achieved when measuring circumferential strain and strain rates, with more variability observed in indices of radial and long axis function. 3D FT-CMR delivered lower values for strain and strain rate compared to 2D analysis. Therefore, normal range values between 2D and 3D feature tracking are not interchangeable.

To our knowledge, there are no other data currently available that determine reproducibility of 3D deformation analysis using feature tracking. In the majority of measurements, there were benefits in terms of reduced intra- and inter-observer variability. Moreover, the data suggest that the best approach in terms of reproducibility is to repeat analysis and average the result, although it is not known whether this delivers incremental clinical merit. It should be remembered that CMR-based feature tracking is subject to considerable inter-vendor variability which is lowest for GCS and can be reduced by averaging with repetitive measurements [15]. The largest improvement in reproducibility can be seen with 3D FT for radial strain and strain rates [9]. Feature tracking in the radial direction is perhaps most sensitive to through-plane feature loss since it is dependent upon the software tracking subtle twist along the endo- and epicardial borders. Unlike the measurement of Ell where through plane loss of the original segment of the mitral annulus is replaced by an adjacent segment of mitral annulus which is positioned identically for continued tracking, the through-plane loss of a subtle myocardial feature along the radial direction results in complete information loss and hence the potential for larger degrees of mistracking.

To our knowledge, there are no other data currently available that compare 2D with 3D deformation analysis using the same FT-CMR package. The results however, mirror the findings of 3D echocardiography which have demonstrated that absolute values are generally lower than those obtained via 2D methods, whether using block matching, elastic registration or model-based analysis techniques [16]. 3D echocardiography for measurement of strain and strain rate however, has been adversely affected by both poor spatial and temporal resolution, leading to coarser speckle patterns and a higher speckle decorrelation between subsequent volumes. Moreover, the need to stitch together volumes to achieve adequate frame rates for analysis at higher heart rates has limited the clinical application of this technique. In contrast to 3D echocardiography, 3D FT-CMR has high feasibility and was possible in all subjects in the current study with the main requirement being a minimum of 25 phases per cine study. Two-dimensional strain analysis is troubled by the through-plane loss of features into the third dimension. As the LV twists during contraction, the out of plane motion of one segment exaggerates the perceived degree of muscle shortening, thereby resulting in the over-estimation of myocardial movement [17]. 3D FT-CMR is able to overcome this limitation and therefore produces lower absolute value strain and strain rates that may be a closer reflection of the underlying myocardial mechanics. This phenomenon mirrors how a normal ventricle can be seen undergoing a 40% reduction in 2D diameter (the transition from end diastole to end systole) with only a 15–20% reduction in actual muscle fibre length [18]. 3D FT-CMR strains correlated with other markers of systolic function including LVEF as well as Ecc and Ell derived from TomTec Diogenes—a previously validated 2D strain analysis software. Although Diogenes produced higher peak strain values compared to 3D FT-CMR on cvi42, this difference was of a similar order to the 2D FT-CMR used in the main study. This difference can be attributed to through-plane feature loss and the previously reported finding that measured strains are higher towards the endocardium [19], although algorithm differences may also contribute. The Diogenes algorithm utilises an optical flow-based tracking technique similar to that of speckle tracking echocardiography [20], meanwhile cvi42 employs a 3D incompressible model-based algorithm that has been previously validated to produce accurate anatomical tracking [12, 13]. Although improved reproducibility makes 3D FT attractive, there remains a need to investigate whether it also delivers incremental clinical value versus 2D myocardial strain analyses.

No difference was found in our study in strain between genders, which replicates findings from a large meta-analysis that included 2D echocardiography studies [21]. By contrast, small sex related differences in strain were described in a 3D echocardiography study of 303 healthy subjects; however, these were considered sufficiently small to be clinically irrelevant and not worthy of producing sex-specific reference ranges [22]. The same study identified a weak relationship between strain, strain rate and age which was also considered too weak to be of clinical significance. Likewise, our study documented a weak relationship between strain, strain rate and age that was directionally different, and that showed a small increase from 50 years. It is possible that this weak relationship reflects the smaller sample size of the deciles within our population in our study and may be an issue with sampling, as no disparities were found either between sexes in conventional measures of indexed ventricular size or function in our study [23].

Segmental strain analysis has been proposed as a useful method for the diagnosis of regional myocardial disease, for example ischaemia and viability. In a study comparing the diagnostic accuracy of 1D, 2D and 3D strain in a porcine model of myocardial infarction, 3D strain provided incremental diagnostic information when delineating dysfunctional and non-viable myocardium compared to 1D or 2D methods of strain analyses [24]. We have provided segmental GCS reference ranges based upon the modified 16-segment AHA model and have demonstrated that the majority of basal and mid-ventricular segments have good reproducibility but at the apex, reproducibility is poor. This effect is likely due to the thinning of the ventricular wall and increased blurring of the endocardium-blood pool boundary. Reproducibility for segmental GLS and GRS is poor and we do not currently recommend these techniques for routine clinical use.

### Limitations

While the clinical utility of 2D strain is well supported in the literature, our data demonstrating lower 3D values have only been acquired in a normal population; it is therefore not yet possible to determine whether 3D FT-CMR will provide incremental value in disease cohorts. While recent data have emphasised the incremental value of 2D deformation analysis on echocardiography across a range of populations and cardiovascular disease, including subjects from the community with preserved and impaired ventricular function [25], the clinical benefit of 3D analysis on feature-tracking has yet to be explored. In theory, the ability to measure true 3D myocardial motion should provide a better view of myocardial mechanics, with improved reproducibility, in comparison to echocardiography which produces a composite measure of GLS from 2D images in the apical four chamber, two chamber and long axis. Further research is needed to compare the relative clinical value of 2D and 3D FT-CMR in disease states.

We have not recorded the time taken for each of our FT-CMR analyses. However, we feel that time requirement is not an important factor to distinguish between 2D and 3D FT-CMR as on a practical basis, the same contouring used for volumetric CMR analyses can be recycled for feature tracking.

We have included 20 subjects per decile of age for generation of normal ranges. While numbers can be larger, this sample set-up mirrors that of previous reference range studies [9]. Furthermore, given the minor effect age imposes on strain and strain rates, the results have been presented as a single cohort.

## Conclusions

In summary, 3D FT-CMR has superior reproducibility compared to its 2D equivalent. Reference ranges for myocardial strain and strain rates are provided, demonstrating that 3D FT-CMR derives lower normal values than 2D FT-CMR. While 3D FT-CMR correlates with other markers of systolic function, further work is needed to clarify whether there is incremental clinical benefit from the third dimension compared to 2D FT-CMR.

### Electronic supplementary material

Below is the link to the electronic supplementary material.

Supplementary material 1 (DOCX 28 KB)

Supplementary material 2 (DOCX 38 KB)

## Appendix 1: 2D deformable model

Let r be the position of a point in the reference frame and R(r) its position in the current frame. In order to define the mapping from the reference to the current frame, we work in curvilinear coordinates with respect to the mid-curve.

Let m(u) = [x(u), y(u)] represent the mid-curve in the reference frame. The curve is in parametric form with u being the parameter. Let n ^ (u) represent a unit vector normal to the mid-curve at point m(u). Let γ represent distance from point m(u) in direction n ^ (u). Thus, a point r can be defined by a pair of numbers (u, γ) (curvilinear coordinates) by:

$${\text{r}}({\text{u}},\upgamma )={\text{m}}({\text{u}})+\upgamma \,{{\text{n}}^\Lambda }({\text{u}})$$

Let M(u) = (X(u), Y(u)) represent the mid-curve point in the current frame corresponding to the point m(u) in the reference frame (note that the two have the same parameter u). The point in the current frame corresponding to point r(u, γ) in the reference frame is given by:

$${\text{R}}({\text{u}},\upgamma )={\text{M}}({\text{u}})+\Gamma ({\text{u}},\upgamma ){{\text{N}}^\Lambda }({\text{u}})$$

where ^ N(u) is a unit vector normal to the mid-curve at point M(u) and Γ(u, γ) is the distance of point R(u, γ) to the mid-curve.

Imposing the condition of local area preservation leads to an extra equation for Γ(u, γ). The mapping R(r) is determined if the nodes are known in the reference and in the current frame and the equation for Γ(u, γ) has solution.

At any given point, the radial direction is defined by the unit normal n ^ (u). Using the above formulas for mapping and Appendix: Lagrangian strain tensor, the strain in the direction n ^ (u) (radial strain) reads:

$${E_r}=\frac{1}{2}\left[ {{{\left( {\frac{{\partial \Gamma }}{{\partial \gamma }}} \right)}^2} - 1} \right]$$

while the strain in the cross-radial direction:

$${E_c}=\frac{1}{2}\left[ {\frac{{{{\left| {\frac{{dM}}{{du}}+\frac{{\partial \Gamma }}{{\partial u}}\hat {N}+\Gamma \frac{{d\hat {N}}}{{du}}} \right|}^2}}}{{{{\left| {\frac{{dm}}{{du}}+\gamma \frac{{d\hat {n}}}{{du}}} \right|}^2}}} - 1} \right]$$

## Appendix 2: 3D deformable model

Let r be the position of a point in the reference frame and R(r) its position in another frame.

The mapping R(r) is constructed in the following way:

1. We choose in the reference frame, a set of M points placed on the myocardial wall. These points are to be identified as nodes. Their positions are arbitrary but known r_j_ for j = 1,…, M
2. We assume that the displacement field, u(r) = R(r) − r, can be expanded as a linear combination of M scalar basis functions centered at the nodes positions in reference frame r_j_ each weighted by a coefficient c_j_. For the scalar basis functions we use a radial basis function $f(r)={e^{\frac{{ - {{\left| r \right|}^2}}}{{2{\alpha ^2}}}}}$ centered at the nodes r_j_, for j = 1,…, M, where α controls how fast the function decays. Thus, the mapping is given by the formula:

$${\text{R}}({\text{r}})={\text{r+}}\sum\limits_{{j=1}}^{M} {f({\text{r}} - {r_j}){C_j}}$$

The mapping is determined once the coefficients, c_j_ are determined. Note that if we know the coefficients in one frame, the nodes positions are also known by the above equation. Vice-versa is also true: if we know the nodes positions in a frame, the formula for R(r) becomes a linear system of 3 M equations and 3 M unknowns for the coefficients c_j_. This equation allows us to compute directly the deformation gradient tensor and the Lagrangian strain tensor using the formulas from Lagrangian strain tensor.

### Lagrangian strain tensor

A general deformation of a body can be expressed in the form of a mapping R(r), where r = r(x, y, z) and R = R(X, Y, Z) represent the coordinates in the reference and in the current configurations, respectively. The gradient of this deformation map is called the deformation gradient tensor F:

$${\text{in 2D:}}\quad F=\left[ {\begin{array}{*{20}{c}} {\frac{{\partial X}}{{\partial x}}}&{\frac{{\partial X}}{{\partial y}}} \\ {\frac{{\partial Y}}{{\partial x}}}&{\frac{{\partial Y}}{{\partial y}}} \end{array}} \right];\quad {\text{in 3D:}}\quad F=\left[ {\begin{array}{*{20}{c}} {\frac{{\partial X}}{{\partial x}}}&{\frac{{\partial X}}{{\partial y}}}&{\frac{{\partial X}}{{\partial z}}} \\ {\frac{{\partial Y}}{{\partial x}}}&{\frac{{\partial Y}}{{\partial y}}}&{\frac{{\partial Y}}{{\partial z}}} \\ {\frac{{\partial Z}}{{\partial x}}}&{\frac{{\partial Z}}{{\partial y}}}&{\frac{{\partial Z}}{{\partial z}}} \end{array}} \right]$$

The Lagrangian strain tensor, E is defined by:

$$E=\frac{1}{2}\left( {{F^T}F - I} \right)$$

where F is the deformation gradient tensor and I is the identity matrix. The Lagrangian strain in the direction v ^, where v ^ is a unit vector, is:

$$s(\hat {v})={\hat {v}^T}E\hat {v}.$$
