# Supplementary material for: Reference ranges for three-dimensional feature tracking cardiac magnetic resonance: comparison with two-dimensional methodology and relevance of age and gender
Source: Int J Cardiovasc Imaging. 2017 Nov 27;34(5):761–75. doi: 10.1007/s10554-017-1277-x (PMC5889420; doi:10.1007/s10554-017-1277-x)
Supplement: Supplementary file 1 — Supplementary material 1 (DOCX 28 KB) [file 10554_2017_1277_MOESM1_ESM.docx]

**Supplementary Table 1.** 2D **vs.** 3D strains and strain rates for the whole cohort.

|  |  | *2D* | *3D* | *P* |
| --- | --- | --- | --- | --- |
| *Circumferential* | Peak strain (%) | -20.9±3.6 | -17.6±2.6 | <0.001 |
|  | S’ (1/s) | -1.21±0.34 | -0.92±0.19 | <0.001 |
|  | E’ (1/s) | 1.48±0.44 | 0.96±0.22 | <0.001 |
|  | A’ (1/s) | 0.57±0.22 | 0.48±0.16 | <0.001 |
| *Radial* | Peak strain (%) | 47.6±15.4 | 47.4±12.9 | 0.86 |
|  | S’ (1/s) | 3.11±1.72 | 2.93±1.10 | 0.18 |
|  | E’ (1/s) | -3.76±1.40 | -3.04±1.05 | <0.001 |
|  | A’ (1/s) | -0.70±0.32 | -0.68±0.26 | 0.29 |
| *Longitudinal* | Peak strain (%) | -19.8±2.9 | -14.6±2.7 | <0.001 |
|  | S’ (1/s) | -1.16±0.22 | -0.74±0.25 | <0.001 |
|  | E’ (1/s) | 1.08±0.27 | 0.79±0.31 | <0.001 |
|  | A’ (1/s) | 0.74±0.29 | 0.42±0.15 | <0.001 |

Values are mean + SD. P values are derived from paired t-test.

**Supplementary Table 2**. Gender differences in 3D strains and strain rates.

|  |  | *Male* | *Female* | *P* |
| --- | --- | --- | --- | --- |
| *Circumferential* | Peak strain (%) | -17.3±2.4 | -17.9±2.7 | 0.24 |
|  | S’ (1/s) | -0.92±0.20 | -0.92±0.18 | 0.99 |
|  | E’ (1/s) | 0.91±0.21 | 1.00±0.23 | 0.04 |
|  | A’ (1/s) | 0.48±0.14 | 0.47±0.18 | 0.81 |
| *Radial* | Peak strain (%) | 45.9±12.7 | 49.0±11.9 | 0.21 |
|  | S’ (1/s) | 2.91±1.28 | 2.96±0.90 | 0.81 |
|  | E’ (1/s) | -2.80±0.89 | -3.29±1.14 | 0.02 |
|  | A’ (1/s) | -0.67±0.21 | -0.68±0.31 | 0.85 |
| *Longitudinal* | Peak strain (%) | 14.3±2.8 | 15.0±2.3 | 0.20 |
|  | S’ (1/s) | -0.72±0.28 | -0.75±0.22 | 0.54 |
|  | E’ (1/s) | 0.74±0.28 | 0.84±0.33 | 0.09 |
|  | A’ (1/s) | 0.42±0.14 | 0.42±0.16 | 0.91 |

P values are derived from independent samples t-test.

**Supplementary Table 3.** Intra-class correlation for average of 2 strain rate measurements

|  | ICC average measures | |
| --- | --- | --- |
| 3D GCS | *Intra-observer* | 0.94 (0.91 to 0.96) |
|  | *Inter-observer* | 0.94 (0.89 to 0.97) |
| 2D GCS | *Intra-observer* | 0.90 (0.85 to 0.93) |
|  | *Inter-observer* | 0.79 (0.62 to 0.89) |
| 3D GCS S’ | *Intra-observer* | 0.90 (0.84 to 0.93) |
|  | *Inter-observer* | 0.80 (0.64 to 0.89) |
| 2D GCS S’ | *Intra-observer* | 0.62 (0.43 to 0.74) |
|  | *Inter-observer* | 0.56 (0.24 to 0.77) |
| 3D GCS E’ | *Intra-observer* | 0.78 (0.68 to 0.85) |
|  | *Inter-observer* | 0.84 (0.70 to 0.91) |
| 2D GCS E’ | *Intra-observer* | 0.43 (0.15 to 0.62) |
|  | *Inter-observer* | 0.01 (-0.74 to 0.44) |
| 3D GCS A’ | *Intra-observer* | 0.96 (0.95 to 0.98) |
|  | *Inter-observer* | 0.96 (0.94 to 0.98) |
| 2D GCS A’ | *Intra-observer* | 0.85 (0.77 to 0.90) |
|  | *Inter-observer* | 0.74 (0.52 to 0.86) |
| 3D GRS | *Intra-observer* | 0.90 (0.86 to 0.94) |
|  | *Inter-observer* | 0.88 (0.76 to 0.94) |
| 2D GRS | *Intra-observer* | 0.85 (0.78 to 0.90) |
|  | *Inter-observer* | 0.60 (0.27 to 0.78) |
| 3D GRS S’ | *Intra-observer* | 0.860 (0.79 to 0.91) |
|  | *Inter-observer* | 0.85 (0.71 to 0.92) |
| 2D GRS S’ | *Intra-observer* | 0.73 (0.59 to 0.82) |
|  | *Inter-observer* | 0.24 (-0.28 to 0.56) |
| 3D GRS E’ | *Intra-observer* | 0.80 (0.70 to 0.87) |
|  | *Inter-observer* | 0.67 (0.36 to 0.81) |
| 2D GRS E’ | *Intra-observer* | 0.59 (0.39 to 0.73) |
|  | *Inter-observer* | 0.21 (-0.22 to 0.51) |
| 3D GRS A’ | *Intra-observer* | 0.92 (0.88 to 0.95) |
|  | *Inter-observer* | 0.93 (0.86 to 0.96) |
| 2D GRS A’ | *Intra-observer* | 0.78 (0.67 to 0.85) |
|  | *Inter-observer* | 0.82 (0.68 to 0.90) |
| 3D GLS | *Intra-observer* | 0.87 (0.79 to 0.91) |
|  | *Inter-observer* | 0.85 (0.73 to 0.92) |
| 2D GLS | *Intra-observer* | 0.79 (0.69 to 0.86) |
|  | *Inter-observer* | 0.83 (0.68 to 0.60) |
| 3D GLS S’ | *Intra-observer* | 0.46 (0.21 to 0.64) |
|  | *Inter-observer* | 0.76 (0.57 to 0.87) |
| 2D GLS S’ | *Intra-observer* | 0.65 (0.47 to 0.76) |
|  | *Inter-observer* | 0.76 (0.57 to 0.87) |
| 3D GLS E’ | *Intra-observer* | 0.47 (0.21 to 0.64) |
|  | *Inter-observer* | 0.70 (0.46 to 0.83) |
| 2D GLS E’ | *Intra-observer* | 0.69 (0.55 to 0.80) |
|  | *Inter-observer* | 0.65 (0.36 to 0.81) |
| 3D GLS A’ | *Intra-observer* | 0.87 (0.81 to 0.92) |
|  | *Inter-observer* | 0.89 (0.80 to 0.94) |
| 2D GLS A’ | *Intra-observer* | 0.79 (0.69 to 0.86) |
|  | *Inter-observer* | 0.70 (0.46 to 0.84) |
