# Supplementary material for: Reference ranges for three-dimensional feature tracking cardiac magnetic resonance: comparison with two-dimensional methodology and relevance of age and gender
Source: Int J Cardiovasc Imaging. 2017 Nov 27;34(5):761–75. doi: 10.1007/s10554-017-1277-x (PMC5889420; doi:10.1007/s10554-017-1277-x)
Supplement: Supplementary file 2 — Supplementary material 2 (DOCX 38 KB) [file 10554_2017_1277_MOESM2_ESM.docx]

**Supplementary Figure 1.** 16 segment model illustrating peak GRS±SD with mean absolute intra-observer bias±SD and ICC.

**2.**

**Strain 33.8±16.8**

***Bias 12.2*±11.8**

***ICC 0.60***

**3.**

**Strain 30.8±13.3**

***Bias 9.6*±11.3**

***ICC 0.53***

**4.**

**Strain 60.6±19.5**

***Bias* 14.7±14.7**

***ICC 0.56***

**5.**

**Strain 75.7±27.6**

***Bias 18.9*±18.0**

***ICC 0.64***

**6.**

**Strain 70.1±25.9**

***Bias 20.3*±19.1**

***ICC 0.55***

**7.**

**Strain 61.6±22.2**

***Bias* 17.8±18.9**

***ICC 0.49***

**8.**

**Strain 39.5±16.2**

***Bias 1*2.2±13.3**

***ICC 0.53***

**9.**

**Strain 27.7±12.1**

***Bias 9.6*±9.7**

***ICC0.52***

**10.**

**Strain 33.3±14.8**

***Bias* 10.5±9.4**

***ICC 0.63***

**11.**

**Strain 42.8±23.5**

***Bias 14.4*±15.2**

***ICC 0.67***

**12.**

**Strain 55.3±27.9**

***Bias* 18.6±25.1**

***ICC 0.53***

**13.**

**Strain 89.1±35.0**

***Bias 25.3*±27.8**

***ICC 0.55***

**14.**

**Strain 68.6±30.4**

***Bias* 17.8±18.7**

***ICC 0.70***

**15.**

**Strain 57.3±25.8**

***Bias 16.2*±20.7**

***ICC 0.59***

**16.**

**Strain 68.4±34.9**

***Bias 30.5*±38.1**

***ICC 0.35***

**1.**

**Strain 63.9±25.3**

***Bias 15.1±15.41***

***ICC 0.69***

**Supplementary Figure 2.** 16 segment model illustrating peak GLS±SD with mean absolute intra-observer bias±SD and ICC.

**2.**

**Strain -13.5±4.2**

***Bias 3.0*±3.4**

***ICC 0.55***

**3.**

**Strain -11.6±4.1**

***Bias 2.8*±2.4**

***ICC 0.67***

**4.**

**Strain -10.2±5.2**

***Bias 3.2*±2.6**

***ICC 0.73***

**5.**

**Strain -12.7±5.7**

***Bias 4.1*±3.7**

***ICC 0.63***

**6.**

**Strain -12.7±8.7**

***Bias 5.7*±12.7**

***ICC 0.23***

**7.**

**Strain -21.6±2.9**

***Bias 2.1*±1.9**

***ICC 0.63***

**8.**

**Strain -16.8±3.9**

***Bias 2.6*±2.7**

***ICC 0.64***

**9.**

**Strain -16.7±3.4**

***Bias 2.0*±1.8**

***ICC0.74***

**10.**

**Strain -19.4±3.8**

***Bias* 1.8±2.1**

***ICC 0.77***

**11.**

**Strain -20.3±4.3**

***Bias 2.3*±2.5**

***ICC 0.73***

**12.**

**Strain -20.6±3.8**

***Bias 2.7*±2.9**

***ICC 0.58***

**13.**

**Strain -15.6±3.6**

***Bias 2.9*±2.5**

***ICC 0.56***

**14.**

**Strain -15.4±6.1**

***Bias 4.4*±4.9**

***ICC 0.55***

**15.**

**Strain -18.8±5.3**

***Bias 3.8*±4.1**

***ICC 0.57***

**16.**

**Strain -16.2±10.6**

***Bias 3.7*±4.1**

***ICC 0.87***

**1.**

**Strain -10.4±7.4**

***Bias 5.3*±6.8**

***ICC 0.50***
